# Two point-of-care test-based approaches for the exclusion of deep vein thrombosis in general practice: a cost-effectiveness analysis

**DOI:** 10.1186/s12875-023-01992-z

**Published:** 2023-02-07

**Authors:** J. S. Heerink, J. Nies, H. Koffijberg, R. Oudega, M. M. A. Kip, R. Kusters

**Affiliations:** 1 Department of Health Technology and Services Research, Technical Medical Centre, University of Twente, Enschede, the Netherlands; 2 Department of Clinical Chemistry and Haematology, Jeroen Bosch Hospital, Henri Dunantstraat 1, 5223 GZ ‘s-Hertogenbosch, the Netherlands; 3 GGD Twente, Enschede, the Netherlands

**Keywords:** Deep vein thrombosis (DVT), Cost-effectiveness analysis, Point-of-care test (POCT), General practitioner (GP), General practice, D-dimer, Clinical decision rule (CDR), DVT care pathway

## Abstract

**Background:**

In the diagnostic work-up of deep vein thrombosis (DVT), the use of point-of-care-test (POCT) D-dimer assays is emerging as a promising patient-friendly alternative to regular D-dimer assays, but their cost-effectiveness is unknown. We compared the cost-effectiveness of two POCT-based approaches to the most common, laboratory-based, situation.

**Methods:**

A patient-level simulation model was developed to simulate the diagnostic trajectory of patients presenting with symptoms of DVT at the general practitioner (GP). Three strategies were defined for further diagnostic work-up: one based on current guidelines (‘regular strategy’) and two alternative approaches where a POCT for D-dimer is implemented at the 1) phlebotomy service (‘DVT care pathway’) and 2) GP practice (‘fast-POCT strategy’). Probabilities, costs and health outcomes were obtained from the literature. Costs and effects were determined from a societal perspective over a time horizon of 6 months. Uncertainty in model outcomes was assessed with a one-way sensitivity analysis.

**Results:**

The Quality-Adjusted Life Years (QALYs) scores for the three DVT diagnostic work-up strategies were all around 0.43 across a 6 month-time horizon. Cost-savings of the two POCT-based strategies compared to the regular strategy were €103/patient for the DVT care pathway (95% CI: -€117–89), and €87/patient for the fast-POCT strategy (95% CI: -€113–67).

**Conclusions:**

Point-of-care-based approaches result in similar health outcomes compared with regular strategy. Given their expected cost-savings and patient-friendly nature, we recommend implementing a D-dimer POCT device in the diagnostic DVT work-up.

**Supplementary Information:**

The online version contains supplementary material available at 10.1186/s12875-023-01992-z.

## Background

Annually, 1–2 per 1000 persons suffer from deep vein thrombosis (DVT) making it the third most prevalent cardiovascular disease according to an extensive Canadian study [1, 2]. Deep vein thrombosis is associated with short and long-term morbidity and mortality, and recurrence of DVT episodes is common. Severe complications such as pulmonary embolism (PE) or post-thrombotic syndrome (PTS) can occur [3, 4]; these are known to reduce quality of life [5, 6]. Hence, appropriate disease detection is vital to timely initiating effective treatment possibilities. These include oral anticoagulation, in some cases preceded by heparin injections, and compression stockings, depending on the symptoms and other patient characteristics.

As DVT cannot be discriminated from other diseases based on clinical judgment alone, in the Netherlands a specific diagnostic DVT work-up is initiated when a general practitioner (GP) suspects a DVT. A clinical decision rule (CDR) is initially applied to discriminate low (CDR < 4) from high (CDR ≥4) risk patients. This CDR uses a list of predefined clinical criteria, adding one or two points for the presence of each item on this checklist to calculate the final CDR score (Table 1).

**Table 1:** Clinical decision rule for deep vein thrombosis according to the Dutch Committee of General Practitioners [7]

	Risk factors	Weight
**1.**	Male gender	1
**2.**	Use of systemic estrogens (such as contraceptive pills, hormone rings/patches or needles)	1
**3.**	Presence of malignancy	1
**4.**	Surgery in the last month	1
**5.**	Absence of trauma explaining swelling in the calf	1
**6.**	Expanded veins of the limb	1
**7.**	Difference in maximal calf size ≥3 cm	2

High-risk patients immediately undergo a compression ultrasonography (CUS) in the hospital, while a laboratory test (D-dimer) is performed in low-risk patients. A negative (non-elevated) D-dimer rules out DVT, obviating the need for performing a relatively expensive CUS [8–10]; this assumption is supported by the fact that 98.6% of patients with a low CDR score and a negative D-dimer result do not develop a DVT within 3 months [8].

Deviations from this approach occur in current practice for a number of reasons. A recent publication reported that a CDR is correctly applied by 79.3% of Dutch GPs; a plausible explanation why ≈20% of Dutch GPs deviate from this rule could include the widespread use of empirical probability estimates (‘gestalt’) as an alternative for CDRs [11, 12]. Sensitivity of gestalt as a screening tool appears to be similar, but the specificity is substantially decreased compared to a CDR-based approach, leading to unnecessary referrals and an increase in health care costs [13, 14]. Another deviation to the standard approach arises when results of a laboratory D-dimer assay are expected to be received after GP working hours. In these cases, patients are referred directly to the radiology or emergency department, causing unnecessary health care expenditures and discomfort of patients [15].

These undesirable phenomena could be avoided by introducing a point-of-care test (POCT) for D-dimer analysis carried out on low-risk patients. Point-of-care devices can be located at a phlebotomy service to accelerate diagnostic work-up. Patients with positive (elevated) D-dimer results are directly referred to undergo a CUS, while patients with a low CDR and negative POCT D-dimer test are discharged without further involving the GP. As an upcoming alternative, novel quantitative capillary (whole-blood) POCT D-dimer assays could be positioned in the GP practice instead of the traditional phlebotomy service, requiring only a finger-prick instead of a venipuncture, providing results in less than 15 minutes [16]. To our knowledge, the impact of both POCT strategies on health outcomes and costs, remains unknown. We therefore assessed the cost-effectiveness of two POCT-based approaches, comparing them to the most common, laboratory-based, situation in the Netherlands.

## Methods

### Model description

We developed a retrospective patient-level simulation model in Microsoft Excel (version 16.0.14228.20288). It represents the diagnostic pathways of patients with symptoms of DVT from the moment of presentation at the GP until the (correct) diagnosis is established. We applied this health-economic model to analyze the cost-effectiveness of two POCT-based approaches compared to the most common, laboratory-based, situation. Data for the input parameters was obtained from the literature. The outcome measures are the incremental costs, the incremental health effects expressed in Quality-Adjusted Life Years (QALYs), and if these are non-negligible, the incremental cost-effectiveness ratio (ICER) for the two POCT-based approaches compared to the laboratory-based situation.

#### Diagnostic strategies

**Fig. 1:**
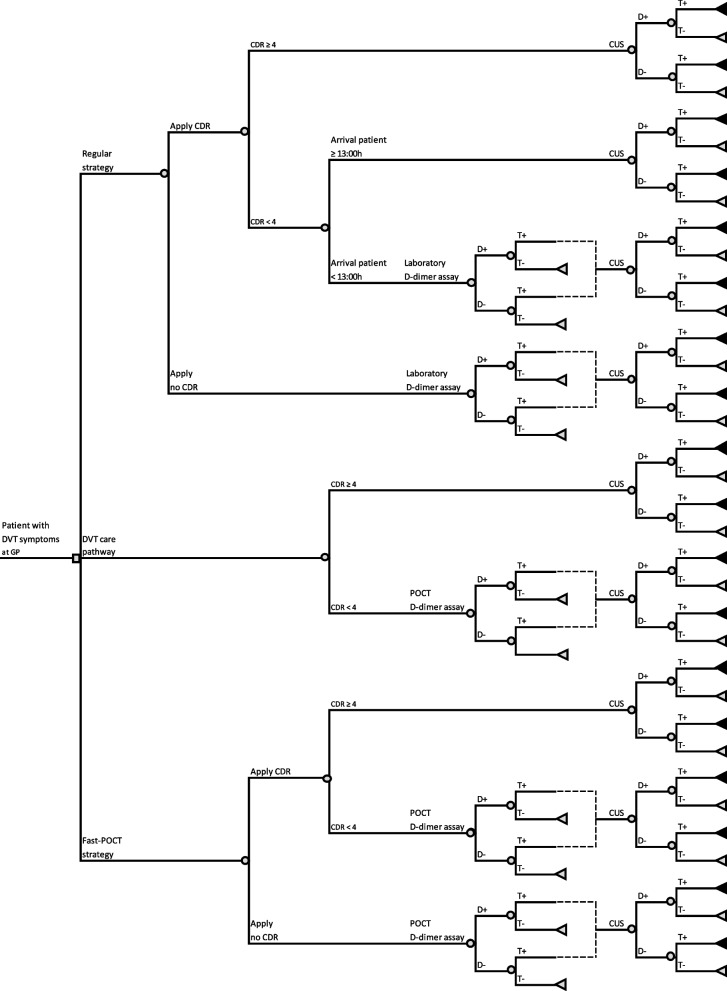
Schematic overview of diagnostic strategies of suspected deep venous thrombosis. CDR = clinical decision rule, CUS: compression ultrasonography, D+ = Disease present, D- = Disease absent, DVT = Deep venous thrombosis, GP = General practitioner, POCT = Point-of-care test, T+ = test with positive result after D-dimer assay or CUS, T- = negative test result after D-dimer assay or CUS

Three different strategies of diagnosing DVT in primary care in the Netherlands were defined. An overview of these strategies is given in Fig. 1.

The regular strategy -the laboratory-based situation- is based on the Dutch College of General Practitioners policy guidelines. These recommend that a CDR and a subsequent quantitative D-dimer assay should be used to rule out DVT [17]. In addition, we took into consideration whether the laboratory D-dimer result would be received during normal GP business hours. If so, only in case of a positive D-dimer would the patient be referred to undergo a CUS, as DVT is directly ruled-out if the D-dimer result is negative. However, should the result not be in time, the patient would be directly referred to undergo a CUS without a preceding laboratory D-dimer assay.

In an alternative approach, the DVT care pathway, patients are referred to the phlebotomy service at the hospital if, based on their CDR score, they are categorized as low risk [18]. A quantitative D-dimer assay is then performed on a POCT device present at the phlebotomy service. If the D-dimer results are positive, patients will directly undergo a CUS. Accordingly, the radiologist will, depending on the results, refer the patient to the internist without intervention of the GP. Those with a negative D-dimer are discharged and left to the attention of the GP.

A third strategy involves the introduction of a POCT D-dimer device at the GP practice; this approach is referred hereafter as the fast-POCT strategy. If the patient is categorized as low-risk according to the CDR score, the GP determines the patient’s D-dimer value during the consultation. If the D-dimer value is positive, the patient is referred to undergo a CUS; if negative, the patient is discharged.

In all three strategies GPs can deviate from the current guidelines and may or may not apply the CDR. Given that some GPs may choose not to use a CDR, we also accounted for this scenario. In practice, as part of the current treatment protocol, a CDR should be used for every patient entering the DVT care pathway.

### Model inputs

Of patients presenting at the GP with suspected DVT, 38.4% are male with an average age of 56.9 [8, 10, 19, 20]. In our model we assumed that 79.3% of GPs correctly apply a CDR [11].^1^ On the basis that patients arriving after 13:00 h are directly referred for a CUS, we assumed that 70.8% of patients would arrive in time for a laboratory D-dimer assay [21–24]. The probabilities of a positive or negative D-dimer test or CUS result were obtained from multiple studies [8, 14, 19, 20, 25–27]. A weighted estimate of sensitivity and specificity was obtained from a series of POCT and laboratory D-dimer assays in order to get a representative performance estimate of these two D-dimer assays [16, 25, 28–31]. The impact of a false negative test result, in terms of complications (PE and PTS), as well as accompanying costs and health impacts were included in the model, as were complications due to incorrectly treating patients with false positive D-dimer test results (major bleeding). A six-month time-horizon was used in this model; following an initial DVT episode, a three-month treatment window is recommended, so another 3 months later, we expected only few new complications to occur in both treated and untreated patients that could be traced back to the original DVT [7].

Quality adjusted life years were used as effectiveness outcome. The health states incorporated in the model were: healthy, suffering from DVT, suffering from DVT complicated by PTS, PE, major bleeding or recurrence [5, 6, 32–34]. As a patient can only have a maximum of one quality of life score per year, the maximum per half year, and thus the upper limit of the 95% CI, was set at 0.5.

In accordance with the Dutch costing manual, we considered all costs from a societal perspective. These start from the moment a patient arrives at a GP until (correct) diagnosis up to 6 months of anticoagulant treatment or the occurrence of potential complications. Costs of both office and phone consultations were obtained from the Dutch costing manual [35]. Diagnostic costs from a blood draw, a laboratory D-dimer test and a CUS were derived from the Dutch Health Care Authority [21]. Costs per D-dimer POCT test were determined by considering procurement costs of the device and price per test (including kits and disposables) and were retrieved from sales brochures and retailers. We also included travel and parking costs [21]. The number of production hours lost due to (diagnosing) DVT and potential complications were derived from literature and were both based on age group, gender and the net labor participation [17, 32, 36, 37]. These were then multiplied by costs per production hour lost determined based on the net labor participation classified per gender and age group of Dutch society [17]. To determine treatment costs and costs of potential complications, we derived an average from the literature [21, 38–41]. The cost estimation was then converted to 2021 Euros based on the Dutch consumer price index [42].

An overview of the model input parameters for each of the three patient strategies used in the model, costs and quality of life score (i.e., utility values) with their corresponding CIs can be found in Tables 2, 3 and 4, respectively. Assumptions for the model are provided as supplementary material.

**Table 2:** Model input parameters for strategies used, showing the parameter, the value (probability) used in the model, the 95% CI interval, the distribution used and the data source

Model input parameters	Category	Probability	Lower limit 95% CI	Upper limit 95% CI	Distribution	Reference
**Patient Demographics**	Sex (male)	38.4%	37.0%	39.8%	Beta	[8, 10, 20, 43]
**Age group**	< 34	9.8%	8.9%	10.6%	Dirichlet	[8, 10, 20, 43]
	35–44	14.3%	13.3%	15.4%	Dirichlet	[8, 10, 20, 43]
	45–54	21.5%	20.3%	22.7%	Dirichlet	[8, 10, 20, 43]
	55–64	26.9%	25.7%	28.2%	Dirichlet	[8, 10, 20, 43]
	65–75	11.9%	11.0%	12.8%	Dirichlet	[8, 10, 20, 43]
	> 75	15.5%	14.5%	16.5%	Dirichlet	[8, 10, 20, 43]
**Probability DVT**	Incidence DVT	15.8%	14.2%	17.4%	Beta	[12, 25, 43]
**CDR**	GP applies CDR	79.3%	76.7%	81.8%	Beta	[11]
	CDR ≥4	42.6%	40.4%	44.8%	Beta	[12, 25, 43]
**Arrival patient at GP**	Arrival patient before 13:00 h	70.8%	33.1%	100.0%	Beta	[1, 22–24, 44]
**Regular strategy (CDR not applied)**	Incidence DVT regular CUS	15.8%	14.2%	17.4%	Beta	[12, 25, 43]
	Sensitivity regular CUS	93.8%	81.9%	100.0%	Beta	[12, 25, 43]
	Specificity regular CUS	97.8%	94.7%	100.0%	Beta	[12, 25, 43]
	Incidence DVT regular D-di	15.8%	14.2%	17.4%	Beta	[12, 25, 43]
	Sensitivity regular D-di	97.9%	90.9%	100.0%	Beta	[12, 25, 43]
	Specificity regular D-di	54.2%	43.6%	64.9%	Beta	[12, 25, 43]
	Incidence DVT regular CUS after D-di	28.6%	16.5%	40.6%	Beta	[12, 25, 43]
	Sensitivity regular CUS after D-di	93.8%	81.8%	100.0%	Beta	[12, 25, 43]
	Specificity regular CUS after D-di	97.8%	93.2%	100.0%	Beta	[12, 25, 43]
**POCT-based approaches (CDR not applied)**	Incidence DVT CUS	35.7%	21.1%	50.4%	Beta	[12, 25, 43]
	Sensitivity CUS	93.8%	81.5%	100.0%	Beta	[12, 25, 43]
	Specificity CUS	97.8%	92.2%	100.0%	Beta	[12, 25, 43]
	Incidence DVT D-di	15.8%	14.2%	17.4%	Beta	[12, 25, 43]
	Sensitivity D-di	93.2%	80.5%	100.0%	Beta	[12, 25, 43]
	Specificity D-di	68.6%	58.7%	78.5%	Beta	[12, 25, 43]
**Regular strategy (CDR applied)**	Incidence DVT > 13:00 h CUS	3.8%	2.7%	4.9%	Beta	[12, 25, 43]
	Sensitivity > 13:00 h CUS	93.8%	69.5%	100.0%	Beta	[12, 25, 43]
	Specificity > 13:00 h CUS	97.8%	94.9%	100.0%	Beta	[12, 25, 43]
	Incidence DVT < 13:00 h D-di	3.8%	2.7%	4.9%	Beta	[12, 25, 43]
	Sensitivity < 13:00 h D-di	97.9%	83.7%	100.0%	Beta	[12, 25, 43]
	Specificity < 13:00 h D-di	54.2%	44.3%	64.2%	Beta	[12, 25, 43]
	Incidence DVT < 13:00 h CUS after D-di	7.8%	6.2%	9.3%	Beta	[12, 25, 43]
	Sensitivity < 13:00 h CUS after D-di	93.8%	69.3%	100.0%	Beta	[12, 25, 43]
	Specificity < 13:00 h CUS after D-di	97.8%	93.5%	100.0%	Beta	[12, 25, 43]
**POCT-based approaches (CDR applied)**	Incidence DVT POCT D-di	3.8%	2.7%	4.9%	Beta	[12, 25, 43]
	Sensitivity POCT D-di	93.2%	68.0%	100.0%	Beta	[12, 25, 43]
	Specificity POCT D-di	68.6%	59.3%	77.9%	Beta	[12, 25, 43]
	Incidence DVT CUS after POCT D-di	10.5%	0.1%	20.8%	Beta	[12, 25, 43]
	Sensitivity POCT CUS after POCT D-di	93.8%	68.7%	100.0%	Beta	[12, 25, 43]
	Specificity POCT CUS after POCT D-di	97.8%	92.6%	100.0%	Beta	[12, 25, 43]
	Incidence CUS after > 4 CDR	31.5%	28.4%	34.7%	Beta	[12, 25, 43]
	Sensitivity CUS after > 4 CDR	93.8%	69.5%	100.0%	Beta	[12, 25, 43]
	Specificity CUS after > 4 CDR	97.8%	94.9%	100.0%	Beta	[12, 25, 43]
**Complications**	PE	27.0%	13.6%	40.4%	Beta	[8]
	PTS	6.0%	3.1%	9.0%	Beta	[45]
	Major Bleeding	3.5%	1.8%	5.2%	Beta	[46, 47]
	Recurrence	5.0%	2.6%	7.5%	Beta	[48]

*DVT* Deep venous thrombosis, *CDR* Clinical Decision Rule, *CUS* Compression Ultrasonography, *D-di* D-dimer assay, *GP* General practitioner, *POCT* Point-of-care Test, *PE* Pulmonary Embolism, *PTS* Post Thrombotic Syndrome

**Table 3:** Model input parameters for costs, showing the parameter, the value (amount) used in the model, the 95% CI interval, the distribution used and the data source

Cost parameters	Category	Cost	Lower limit 95% CI	Upper limit 95% CI	Distri-bution	Refer-ence
**Consultation**	Appointment at GP practice	€ 36.45	€ 18.60	€ 54.32	Gamma	[35]
	Consult by phone	€ 18.78	€ 9.58	€ 27.98	Gamma	[35]
**Diagnostics**	Blood draw	€ 6.30	€ 3.21	€ 9.39	Gamma	[21]
	D-di POCT at office (fast-POCT strategy)	€ 32.83	€ 16.74	€ 48.90	Gamma	Company info
	D-di POCT including AQT (DVT care pathway)	€ 8.83	€ 4.50	€ 13.15	Gamma	Company info
	D-di assay on STA-Liatest	€ 5.81	€ 2.96	€ 8.65	Gamma	[21]
	CUS	€ 92.19	€ 47.02	€ 137.37	Gamma	[21]
**Travel**	To GP	€ 0.23	€ 0.11	€ 0.34	Gamma	[35]
	To phlebotomy service	€ 4.79	€ 2.44	€ 7.13	Gamma	[35]
**Treatment**	Treatment costs	€ 580.79	€ 269.20	€ 865.37	Gamma	[21, 38, 39, 41]
**Complication**	Cost PE	€ 4501.04	€ 2259.53	€ 6706.54	Gamma	[21, 38–41]
	Cost PTS	€ 4001.12	€ 2040.57	€ 5961.67	Gamma	[21, 40, 41]
	Cost major bleeding	€ 7174.99	€ 3659.25	€ 10,690.73	Gamma	[21, 38–41]
	Cost recurrence	€ 673.78	€ 346.38	€ 1012.87	Gamma	[21, 38, 39, 41]
**Male productivity costs per hour**	< 34	€ 30.61	€ 15.61	€ 45.61	Gamma	[17]
	35–44	€ 37.25	€ 19.00	€ 55.50	Gamma	[17]
	45–54	€ 37.16	€ 18.97	€ 55.38	Gamma	[17]
	55–64	€ 32.88	€ 16.76	€ 48.99	Gamma	[17]
	65–75	€ 7.49	€ 3.82	€ 11.16	Gamma	[17]
	> 75	€ 2.08	€ 1.06	€ 3.10	Gamma	[17]
**Female productivity costs per hour**	< 34	€ 27.57	€ 14.06	€ 41.08	Gamma	[17]
	35–44	€ 30.56	€ 15.59	€ 45.55	Gamma	[17]
	45–54	€ 30.34	€ 15.47	€ 45.21	Gamma	[17]
	55–64	€ 23.81	€ 12.14	€ 35.48	Gamma	[17]
	65–75	€ 3.47	€ 1.77	€ 5.17	Gamma	[17]
	> 75	€ 0.76	€ 0.39	€ 1.13	Gamma	[17]

*DVT* Deep venous thrombosis, *CUS* Compression Ultrasonography, *D-di* D-dimer assay, *GP* General practitioner, *POCT* Point-of-care Test, *PE* Pulmonary Embolism, *PTS* Post Thrombotic Syndrome

**Table 4:** Model input parameters for quality of life, showing the parameter, the value (utility) used in the model, the 95% CI interval, the distribution used and the data source

Quality of life estimates	Category	Utility	Lower limit 95% CI	Upper limit 95% CI	Distri-bution	Refer-ence
**Average quality of life in 6 months**	Healthy	0.4345	0.2163	0.5	Beta	[34]
	Treated DVT	0.3924	0.1961	0.5	Beta	[5, 6]
	DVT with PE	0.3880	0.1940	0.5	Beta	[5, 6, 33]
	DVT with PTS	0.3901	0.1950	0.5	Beta	[5, 6]
	DVT with major bleeding	0.3874	0.1937	0.5	Beta	[5, 6, 46]
	DVT with recurrence	0.3762	0.1883	0.5	Beta	[5, 6]

*DVT* Deep venous thrombosis, *PE* Pulmonary Embolism, *PTS* Post Thrombotic Syndrome

### Probabilistic sensitivity analysis

A probabilistic sensitivity analysis (PSA) was performed by means of a Monte Carlo simulation (10,000 iterations of from 10,000 patients) to demonstrate the effect of joint uncertainty in the input parameters on the outcomes of the model. An incremental cost-effectiveness plane was used to display the uncertainty in model outcomes.

### One-way sensitivity analysis

A one-way sensitivity analysis was performed to determine which parameters substantially influence model outcomes. The impact on the output of each strategy was analyzed by applying the lower and upper limit of the corresponding 95% confidence intervals (CIs) of each parameter in the model. Results are presented in tornado diagrams.

### CHEERS checklist

A checklist based on the Consolidated Health Economic Evaluation Reporting Standards (CHEERS) is provided as supplementary material. This list comprises the items that should be included when reporting economic evaluations of health interventions and describes where to find these items in this publication.

## Results

The average costs per patient were €827 (95% CI: €750–909) for the regular strategy, €724 (95% CI: €656–798) for the DVT care pathway, and €740 (95% CI: €674–812) for the fast-POCT strategy. Average cost-savings for the DVT care pathway and for the fast-POCT strategy compared with the regular strategy were €103/patient (95% CI: -€117–89, a relative reduction of 12.4%) and €87/patient (95% CI: -€113–63, a relative decrease of 10.5%), respectively. As the difference in QALYs was approximately − 0.0002, equal to less than 2 h in full health, this was considered clinically irrelevant. Therefore, no ICER was determined, as the ICER would be unstable with this low denominator. The use of the DVT care pathway instead of the regular strategy could result in health care savings of approximately €2,575,000 half-yearly (€103 * 25,000 patients), thus €5,150,000 annually. The fast-POCT strategy could save approximately €2,175,000 half-yearly (€87 * 25,000 patients), €4,350,000 annually.

**Fig. 2:**
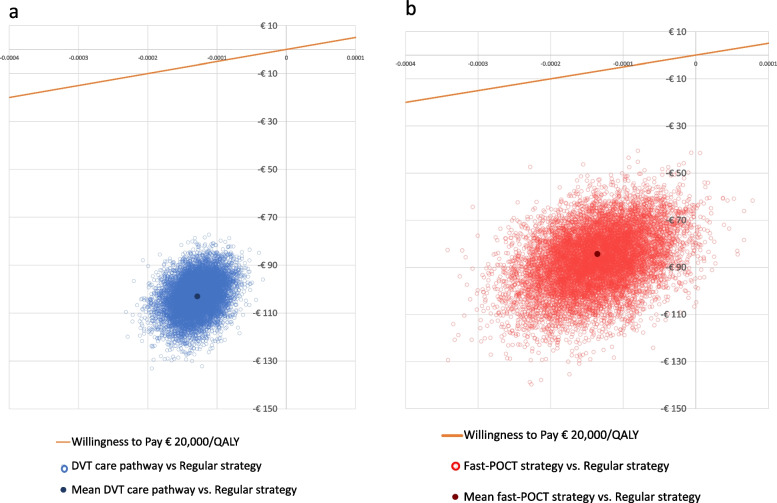
**a** Incremental cost-effectiveness plane of the DVT care pathway versus the regular strategy. **b** Incremental cost-effectiveness plane of the fast-POCT strategy versus the regular strategy

Figure 2a and b show an incremental cost-effectiveness plane, presenting the results of the PSA. These figures show cost savings of 100% when comparing the DVT care pathway and the fast-POCT strategy to the regular strategy

**Fig. 3:**
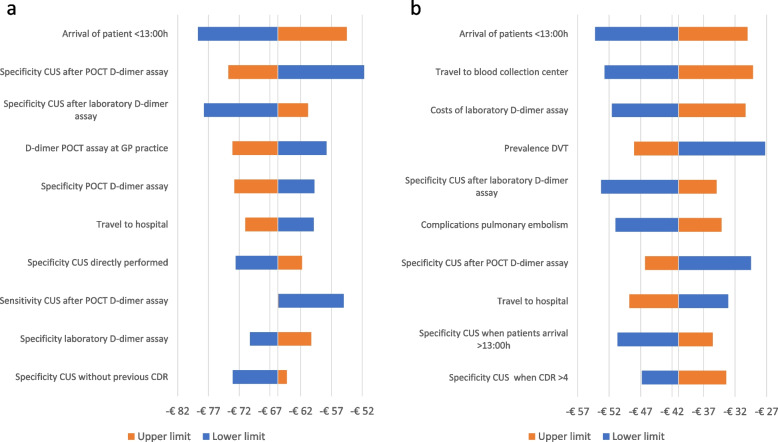
**a** Tornado diagram showing the impact of varying input parameters on model outcome when the regular strategy is compared to the DVT care pathway. *CUS* compression ultrasonography. *POCT* point-of-care. *CDR* clinical decision rule. **b** Tornado diagram showing the impact of varying input parameters on model outcome when the regular strategy is compared to the fast-POCT strategy. *DVT* deep vein thrombosis. *CUS* compression ultrasonography. *POCT* point-of-care. *CDR* clinical decision rule

The tornado diagrams shown in Fig. 3a and b demonstrate the influence of the uncertainties of the input parameters on the incremental costs. Comparing the DVT care pathway and the fast-POCT strategy to the regular strategy shows the time of arrival of patients has the greatest impact on the differences in costs, as more patients are referred directly to undergo a CUS. All outcomes of the univariate sensitivity analyses show that the DVT care pathway and the fast-POCT strategy result in cost-savings compared to the regular strategy.

An additional sensitivity analysis was performed to determine the extent of influence of arrival time of patients at the GP practice on the outcome of the model. Table 5 shows that the arrival time did not influence model outcomes.

**Table 5:** Additional sensitivity analysis for arrival time parameter

Probability patient arriving in time	Incremental effects regular strategy vs. DVT care pathway		Incremental effects regular strategy vs. fast-POCT strategy	
%	€	QALY	€	QALY
0.0	90.92	−0.00019	66.44	−0.00017
70.8	65.49	−0.00017	33.81	−0.00015
100.0	54.52	−0.00015	30.04	−0.00013

## Discussion

We demonstrated that both POCT-based approaches in this study, if implemented, reduce costs from a societal perspective when compared to the regular strategy. The loss of QALYs (< 0.0002 QALYs loss over a six-month time horizon) was considered not to be clinically relevant. In view of these results, using a POCT D-dimer test is the most advantageous option, despite the relatively high costs of POCT devices in terms of purchase and maintenance.

Data from the latest quantitative POCT devices was used in this study. These test systems are considered to be patient and user-friendly as they only require a capillary finger prick of blood, facilitating the application of these devices in settings outside the laboratory [16]. More studies on this topic have been performed on past-generation POCT devices, but none, to our knowledge, have incorporated productivity losses per health state or fully considered production costs and complication losses as detailed as in our study. Neither have they incorporated alternative scenarios like the hybrid approach of a DVT care pathway, or sub-scenarios in which physicians do not follow current guidelines. Our results are in line with earlier studies on now-deprecated POCT devices which demonstrate that POCT-based approaches are less expensive than the regular strategy [40, 41].

In the near future, the rapid evolution of quantitative capillary POCT devices is expected to improve their diagnostic accuracy, further improving health-related outcomes. The false-negative rate is expected to be further reduced with fewer cases being missed. This will lead to a decline of complications in the DVT care pathway and fast-POCT strategy, compared to current model outcomes.

Of note, while a full cost-effectiveness analysis was planned, we focused on reporting results from an economic evaluation (i.e. cost-minimization analysis) due to the fact that differences in QALYs between the three different strategies were very small. These can be explained by the small difference in sensitivity and specificity between the laboratory and POCT-based D-dimer assays.

### Strengths

The main strength of this study is that it was set up to provide an accurate and detailed illustration of diagnostic work-up after a patient with DVT symptoms presents to the GP. In order to achieve this, we incorporated two important real-life input parameters in our model. The first parameter is the estimated production cost loss per health state based on the literature [17, 32, 36, 37]. Net labor participation per complication and associated health-state, sex and age group were used to accurately represent production losses and related costs in real-life DVT patients in the six-month time horizon during and after DVT diagnosis. The second parameter is the arrival time of patients at the GP as this can greatly affect the chosen diagnostic strategy. As these parameters have an impact on costs, they form significant factors.

A further strength is the conservative assumptions made in the model regarding treatment costs, thus possibly underestimating cost savings. For example, treatment and complication costs were based on treatment for 6 months based on available data, even though only 3 months were suggested by the Dutch College of General Practitioners [7]. Moreover, as the sensitivity and specificity of the current POC test devices are lower than their conventional counterparts, more complications were found in the POCT-based approaches. However, D-dimer POCT test devices are rapidly evolving and their diagnostic performance of D-dimer POC tests will most probably improve in the near future; hence, current assumptions about their diagnostic values will probably underestimate the cost-savings of POCT-based strategies. Combining these factors, we expect more cost savings and less health losses using the POCT-based approaches. Furthermore, in our model we assumed that a POC test at the GP practice would lead to a more time-consuming consultation in the fast-POCT strategy. However, if this test could be embedded in the first consultation, this would reduce consultation costs, resulting in additional cost-savings when using the fast-POCT strategy.

### Limitations

Several limitations need to be addressed. First, some input parameters used in the model were obtained from the literature, while others were based on assumptions. One of these assumptions is the time of arrival at the GP of a patient experiencing symptoms that could indicate a DVT. There is no specific data available on how many patients arrive too late for the GP to obtain laboratory D-dimer results before the end of the working day. This is relevant as the follow-up cannot be done on the same day. Because of this, arrival time greatly affects the GP’s strategy so we chose to include this variable in our model. We performed a scenario analysis in which all patients arrived on time vs. another scenario in which they were directly referred to undergo a CUS. This analysis showed no substantial change in model outcomes. Therefore, it is unlikely that this limitation has affected our conclusions.

In other cost-effectiveness studies, the post-DVT mortality risk was included [39, 40]. However, the relatively low incidence of DVT, combined with a low mortality risk and the negligible increase in false negative D-dimer test results due to POCT, would result in a negligible increase in mortality risk. Thereby, including mortality in this already comprehensive and detailed model would not have altered the results in the six-month time-horizon, or when applying a longer time horizon, and would therefore unnecessarily complicate the model.

### Implications for practice

Patient-friendliness is an important factor in health care. We were unable to include patient-friendliness in our model, as no quantitative measures are currently available for this item. However, waiting time can be reduced, resulting from (i) a POCT assay being performed during the GP consultation in the fast-POCT strategy and (ii) direct referral to the radiologist and internist in the DVT care pathway. These both substantially contribute to patient-friendliness. Furthermore, once the consultation is completed in the two POCT-based approaches, GPs are no longer responsible for follow-up of their patients, so this will also reduce the risk of errors.

We demonstrated that both the DVT care pathway and the fast-POCT strategy will save costs when compared to the regular strategy. If a final decision between these two strategies has to be made, the size and location of the GP practice should be taken into consideration. Larger practices in remote areas may benefit more from the fast-POCT strategy as the majority of (mostly negative-D-dimer) patients will not be referred; this would greatly reduce their travel time. Moreover, in reality, per-test prices generally tend to decrease with higher volumes. For the same reason, smaller practices may benefit more from the DVT care pathway.

## Conclusions

In conclusion, both POCT-based approaches showed cost-savings, with a negligible loss of QALYs compared to the most common current strategy. We are convinced that with the demonstrated cost savings and the added patient and user-friendliness, the DVT care pathway and the fast-POCT strategy should be introduced in clinical practice.

## Supplementary Information

**Additional file 1.** Overview of underlying assumptions for the model in our study.

**Additional file 2.** CHEERS Checklist.

## Data Availability

All data generated or analyzed during this study has been included in this published article and its supplementary files.

## Abbreviations

DVT: Deep vein thrombosis
PE: Pulmonary embolism
PTS: Post-thrombotic syndrome
GP: General practitioner
CDR: Clinical decision rule
CUS: Compression ultrasonography
POCT: Point-of-care test
QALY: Quality-adjusted life years
ICER: Incremental cost-effectiveness ratio
PSA: Probabilistic sensitivity analysis

## Footnotes

1 Incorrect application of a CDR includes ‘inappropriate CPR for PE used’, ‘inappropriate patients (already using anticoagulants, pregnant or postpartum, age < 18), ‘incorrect summation of CPR points’, ‘inappropriate use of D-dimer test (unnecessary when CPR > = 4, omitted D-dimer when CPR < = 2)’ and ‘deviation from referral recommendation’.
