# Supplementary material for: Two point-of-care test-based approaches for the exclusion of deep vein thrombosis in general practice: a cost-effectiveness analysis
Source: BMC Prim Care. 2023 Feb 7;24:42. doi: 10.1186/s12875-023-01992-z (PMC9903487; doi:10.1186/s12875-023-01992-z)
Supplement: Supplementary file 1 — Additional file 1. Overview of underlying assumptions for the model in our study. [file 12875_2023_1992_MOESM1_ESM.docx]

### **Supplementary material: overview of underlying assumptions for the model in our study**

### **Assumptions related to GPs decisions and diagnostics**

- When the CDR is 4 or higher, or the results are expected not to get back in time, the patient is immediately referred to undergo a CUS. This was directly done at the radiologist, as the GPs are encouraged to not send the patients to the emergency room.
- Since no information is available whether GPs perform a D-dimer when no CDR is being used, it is assumed that all GPs performed a D-dimer if the CDR was less than 4, or when the CDR was not applied.
- A positive D-dimer assay means that the D-dimer level of the patient exceeds a certain threshold (validated by the specialist) and the patient is still eligible for DVT.
- It is assumed that the working day of a GP is from 08:00h to 17:00h.
- It is assumed that after 13:00h, it was expected that results would be reported too late to send the blood sample to the laboratory for a D-dimer assay, as the results would be received after the GPs working hours. It takes approximately 2-3 hours to be tested and approximately 1 hour to get the results back.
- In the fast-POCT process, it is assumed that a GP always uses the POCT device present at the GP practice, and not the laboratory D-dimer assay.
- It is assumed that all patients that had to go to the hospital, travel with their own transportation.

###

### **Assumptions related to probabilities**

- The percentage of patients arriving before 13:00h was determined by examining GP’s daily routines. (2,3) As GPs in the Netherlands often schedule consultations in the morning between 08:00h and 11:00h, there is 2.75-hour time for consultations per day, 423.7 consultation hours per year as the average GP works 46 weeks with 0.67 FTE. (4,5) 74% of the consultations takes 10 minutes, and 26% is 20 minutes. The mean time per consultation is therefore 12.6 minutes, and 5044.9 consultations could take place between 08:00h-11:00h in one year per practice (2.5 GPs per practice, including a 15-minute break each morning). The total number of consultations per practice per year was 8966. (4) It was assumed that the rest of the consultations (8966 – 5044 = 3922) were evenly spread amongst the other 6 working hours of the GP.
- The diagnostic accuracies of the laboratory and POCT D-dimer assay is assumed to be the same when the CDR is or is not applied.
- The probabilities of testing positive or negative and the associated sensitivity and specificity of the laboratory and POCT D-dimer assay and the CUS were determined using the ‘Health economic decision tree models of diagnostics for dummies: a pictorial primer’ and literature on diagnostic accuracies and prevalence of DVT. (6)
- It is assumed that the diagnosis is set by the results of the CUS.

### **Assumptions related to costs and effects**

- It is assumed that the consultations wherein a D-dimer POCT is performed *in* the GPs office takes up two consultations of the GP.
- The costs for a D-dimer were assumed to be the average of multiple POCTs, obtained from five market companies, and included rent or procurement costs. (7)
- It is assumed that a patient only pays parking costs at the hospital.
- Productivity losses are measured with the friction cost method.
- Cost of the treatment consisted of 50% DOAC treatment, and 50% LMWH treatment. (8,9) Moreover, it is assumed that all patients that tested positive for DVT received a compression stocking of which the costs were included in the total treatment cost.
- It is assumed that false positive patients had the same QALYs as the true positive patients, as they believe they have DVT. They only had a (minor) risk on major bleeding complications.
